# Electromagnetic Non-Resonant Vibration Energy Harvesters with Linear Magnetic Gear

**DOI:** 10.3390/mi17101121

**Published:** 2026-09-25

**Authors:** Junyi Wang, Eun Sok Kim

**Affiliations:** Department of Electrical and Computer Engineering, University of Southern California, Los Angeles, CA 90089, USA; eskim@usc.edu

**Keywords:** energy harvesting, wearables, non-resonant electromagnetic energy conversion, magnetic gears

## Abstract

This paper presents a non-resonant vibration energy harvester (VEH) with an external magnet array to enhance the output voltage through a linear magnetic gear (MG) effect. A magnet array with an optimal number of smaller-sized magnets is fixed above the VEH (2.5 cc and 5.5 g) and is shown to increase the relative velocity between the internal magnet array (suspended by ferrofluid bearings) and the coil array (mounted on the VEH chamber), increasing the time portion of non-zero relative velocity per cycle. The electromagnetic VEH, with a coil array of 2000 turns and 525 Ω, is measured to produce an open-circuit voltage of 26.3–61.6 mVrms over 0.8–4.0 Hz under 0.3 g acceleration.

## 1. Introduction

Vibrational energy harvesters can be used to harvest power from human motion for wearable sensors and actuators [1,2], as the human body is an exceptionally rich and continuous source of mechanical energy [3]. Routine physical activities, including locomotion, joint articulation, heel strike, and limb oscillation, generate substantial kinetic energy, while underlying physiological processes, including myocardial contractions, hemodynamic flow, and gastrointestinal peristalsis, contribute mechanical and thermal energy at the organ level [4]. A large portion of this energy is inevitably consumed in sustaining essential bodily functions; nevertheless, even the fraction that remains unharvested represents a non-trivial energetic resource [5]. This recognition has stimulated considerable and growing research interest in developing energy-harvesting devices capable of capturing biomechanical energy across diverse motion modalities and transducing it into exploitable electrical output [6,7]. However, human motion is predominantly low frequency, generally below 4 Hz, and highly irregular in both amplitude and periodicity, varying substantially across individuals, speeds, and physical conditions [8,9]. This renders the excitation source fundamentally unpredictable and broadband, directly in conflict with conventional resonance-based harvesting mechanisms [8,10,11]. Another challenge in human energy harvesting is maximizing electrical power output while minimizing the additional physical burden on the human [5,6]. Even for a non-resonant VEH based on a ferrofluid bearing [12,13], which is the most promising approach for generating power from humans’ daily motion and activities, the low acceleration at low frequencies of vibration means inherently low available mechanical power. The low-frequency vibration, and therefore low-velocity motion along with sub-g acceleration, of human motions means that we need to enhance the low-velocity and low-frequency motion, in order to avoid a very large and heavy VEH for power generation from humans’ daily activities.

Thus, we have, for the first time, devised the concept of using a linear magnetic gear (MG) to increase power generation from a very low-velocity (and low-frequency) vibration with a minimal impact on the size and weight of our previously published non-resonant VEH, which is composed of a chamber that houses an internal magnet array (suspended by a ferrofluid bearing) and a coil array, but is without any MG [12,13]. A magnetic gear (which uses magnetic forces between two magnet arrays) does not require physical contact, unlike mechanical gears, as it is based on magnetic attraction and repulsion to transfer power. Cylindrical magnetic gears have been used with significant advantages, including minimal acoustic noise and vibration, maintenance-free operation, high transmission efficiency, and inherent physical isolation between the input and output shafts [14,15]. Non-modulated magnetic gears are a class of magnetic transmission devices that operate without ferromagnetic modulation pieces or flux-modulating rings. Unlike field-modulated magnetic gears, which rely on spatial harmonic conversion between different pole-pair numbers, non-modulated magnetic gears transmit force or torque through the direct coupling of permanent magnet arrays without modulation poles. Omitting ferromagnetic modulation poles in non-modulated magnetic gears reduces iron losses, eddy-current effects, and manufacturing complexity [16]. The gear ratio, which is related to the relative sizes of the magnets, can be adjusted by varying the configuration and arrangement of the magnets. Consequently, a magnetic gear offers a unique means to increase the power generation from a non-resonant electromagnetic VEH. This paper describes a non-resonant VEH with a linear magnetic gear optimized for sub-g and sub-Hz accelerations, typically associated with human daily activities, and with its volume and weight arbitrarily limited to 2.5 cc and 5.5 g, respectively, for ease of wearability.

## 2. Methods

The magnetic gear is achieved by placing an array of external magnets above the top of a non-resonant VEH chamber (containing an array of internal magnets) at a certain distance, which is optimized and reduced by partially demagnetizing the external magnets. The different sizes of the two magnet arrays affect the velocity of the internal magnets as they move through the chamber. We chose a smaller-sized magnet as the external magnet to increase the relative velocity between the larger internal magnets and the chamber on which a coil array is attached.

The external magnet array pulls on the internal magnets in the direction of their travel along one direction in an in-plane manner, with no stable equilibrium point involved. As the internal array passes beneath the external array, it experiences a net forward pull force along the traveling direction, unlike the conventional cylindrical gear, which provides rotational force along a cylindrical axis. The velocity enhancement is quantified by observing relative velocity with a high-speed camera and correlating it with the expected gear ratio. We define an equivalent velocity gain, G_v_ = v_r+MG_/v_r_, as the ratio of peak relative velocity, v_r+MG_, with and without the magnetic gear (MG), v_r_, under identical excitation. Since the open-circuit voltage scales with the relative velocity, G_v_ can serve as a measurement-based gear ratio for this non-contact, velocity-amplifying magnetic coupling. The VEH chamber contains an array of four magnets (the number of which depends on the VEH size specification) suspended by ferrofluid bearings and arrays of coils at the chamber’s top and bottom (Figure 1). An external array of smaller-sized magnets, 1/3 the thickness and width of the internal magnets, is fixed at a certain distance above the center of the VEH chamber for a linear magnetic gear. The internal magnets have magnetization directed through their thickness, while the external ones have magnetization directed through their width.

In obtaining Figure 2, an ideal motion of the magnets inside the chamber is assumed, with frictionless motion, completely inelastic collisions, and immediate separation when the velocity of the magnet array differs from that of the chamber. The ferrofluid bearings provide frictionless support for the internal magnet array, and the relative velocity between the magnet array and the coil array (needed for power generation) is non-zero only 21% of the time (for 0.5 Hz, 0.2 g sinusoidal acceleration in a 22 mm movable range chamber), a waste of three-quarters of a period. Until the chamber reaches its maximum velocity, the internal magnet array moves with the chamber. Then, the magnet array departs from the end of the chamber at the maximum chamber velocity (due to frictionless support from the ferrofluid bearing) until it collides with the other end of the chamber. For a given acceleration, as the frequency is increased, the zero relative velocity time (so-called dead zone) is reduced because of the decrease in the maximum chamber velocity, since velocity is acceleration divided by frequency.

At a critical frequency *ω_c_* where the applied vibration amplitude is equal to the movable range in the chamber, the magnet array takes a half cycle to move to the other end, colliding with the chamber, which at that very moment moves in the opposite direction at the maximum velocity [1],

$$\omega_{c}=\sqrt{\frac{\pi a}{L}}$$

where *a* and *L* are the amplitude of applied acceleration and the movable range in the VEH chamber for the internal magnet array, respectively.

If the frequency is increased further while keeping the acceleration constant, the relative motion becomes aperiodic or random. In this case, the internal magnet array’s motion will not have any zero relative velocity time, and it will not reach the maximum velocity due to collisions with the chamber either. Most human motions’ frequency will be less than the critical frequency of the corresponding acceleration, so the dead zone takes a large portion of one cycle of the motions. As acceleration decreases to sub-g levels, as in typical human motion, the critical frequency decreases, limiting the operational bandwidth of the VEH. This limitation cannot be solved by simply shortening the movable range, as it reduces voltage generation by decreasing both the power generation time and the number of coil turns. However, all the limits above on relative movement can be overcome by a linear magnetic gear (MG), since the attractive force between the two magnet arrays can pull the internal magnet array into motion during the time when it would normally reside at the end of the chamber. This phenomenon not only reduces the no-power generation time but also increases the critical frequency.

The linear magnetic gear is modeled in COMSOL Multiphysics 6.0 as two arrays of magnets in air. The COMSOL result in Figure 3 is obtained with a refined mesh setting, *Z*-axis and *X*-axis magnetizations set the same as in the experiments, and a Dirichlet boundary condition. The simulations are to qualitatively illustrate the sign and spatial distribution of the magnetic gear force. All quantitative results presented, including RMS voltage improvement, relative velocity increase, equivalent gear ratio, optimal magnet count, and separation distance, are obtained from direct experimental measurement, independent of the simulation. Four 3.2 (length) × 6.4 (width) × 3.2 (thickness) mm^3^ internal magnets (with their magnetization through their thickness direction, i.e., *z*-axis) are positioned at different locations along the *x*-axis, while an array of 1.0 (length) × 6.4 (width) × 1.0 (thickness) mm^3^ external magnets (with their magnetization through their length direction, i.e., *x*-axis) is stationary in the *x*-axis center, separated from the internal magnet array by 4 mm along the *z*-axis. The smaller external magnets not only influence the magnetic field distribution around the larger internal magnets but also exert a constant force as the internal magnets move beneath them, thereby giving the internal magnets an additional acceleration that increases their velocities (Figure 3).

The simulated force–displacement profile (Figure 3d) is intended to describe why the gear force is directional (forward-pulling) rather than a restoring force as in a magnetic spring, and it connects this qualitatively to the measured relative velocity increase. The simulated forces have not been validated against directly measured magnetic forces, and the model is therefore used only as a qualitative aid to interpreting the mechanism, not as a quantitatively verified predictive tool. The gear ratio and velocity enhancement reported here are instead obtained directly and unambiguously from experimental measurement, which we consider the most reliable and reproducible way to characterize the effect at this stage.

The experimental setup [13], consisting of a linear actuator (Aerotech ACT115DL, Pittsburgh, PA USA, controlled via Soloist™Motion Composer software), produces sinusoidal motion of the VEH chamber and measures the open-circuit voltage directly from the coil array with an oscilloscope (Figure 4). For this study, the relative velocity between the internal magnet array and the chamber is extracted from videos recorded at 1000 fps by a high-speed camera and tracked using Tracker software 6.3.2. Each reported voltage and velocity value is measured with three VEHs built with identical components and is the average of at least five repeated measurements at the same frequency and acceleration setting.

## 3. Results

### 3.1. Magnetic Gears

The performance of the magnetic gear (MG) is optimized with respect to (1) the distance between the magnet arrays and (2) the number of magnets in each magnet array. The number in the internal magnet array is fixed at 4, but the number in the external magnet array is adjusted to achieve optimal results. The external magnets (1.0 × 6.4 × 1.0 mm^3^ N35 customized magnets from Amazing Magnets, Round Rock, TX, USA) are first fixed at a 10 mm distance above the chamber (containing the four internal 3.2 × 6.4 × 3.2 mm^3^ N52 magnets from K&J Magnetics, Inc., Pipersville, PA, USA). The measured open-circuit voltages of the VEH with different numbers of external magnets show that the increase in the root mean square (RMS) voltage is largest with 4 external magnets, when the VEH is moved at 0.4 g acceleration (Figure 5). The 4-external-magnet MG not only increases the RMS voltage but also increases the critical frequency, much higher than 3 Hz for the VEH without an MG. Increasing the number of external magnets strengthens the attractive force that pulls the internal array along the gear’s full travel, thereby increasing peak relative velocity. Beyond four magnets, however, the combined vertical attractive force grows large enough to partially reduce the ferrofluid bearing’s frictionless suspension, increasing friction between the internal array and the chamber wall. Using this 4-external-magnet MG, the RMS voltage increases by 127% at 4 Hz and is expected to be higher at higher frequencies, since the VEH without MG produces less voltage when the frequency exceeds the critical frequency (Figure 6).

Another factor influencing the gear effect is the distance between the external magnets and the internal magnets, as the magnetic field strength is directly related to this distance. A higher magnetic field strength, which is the case when the distance is smaller, exerts stronger attractive or repulsive forces between the external and internal magnets, even in the vertical direction (when the internal magnets move underneath the external magnets), pulling the internal magnet array to the top side of the chamber and thus negating the frictionless suspension by the ferrofluidic bearings. The highly varying vertical force (peaking at the center of the *x*-axis) can trap the internal magnets in a small region under the external magnets, with almost no relative movement between the internal magnet array and the coil array. On the other hand, increasing the separation distance between the external and internal magnets reduces the gear effect as the in-plane magnetic force decreases. For the magnets (four 3.2 × 6.4 × 3.2 mm^3^ internal magnets and four 1.0 × 6.4 × 1.0 mm^3^ external magnets) tested, 10 mm is measured to be the best separation distance for voltage generation among the tested distances (Figure 7). To reduce this distance in order to reduce the size of the VEH with an MG, the magnetic field strength of the external magnets needs to be lower, and we partially demagnetize the N35 grade magnets by heating the magnets at 180 °C for an hour. The measured RMS voltage indicates that the optimal distance with the weakened magnets is reduced to 6 mm (Figure 8) without sacrificing the RMS voltage. The flux density and long-term stability of the partially demagnetized magnet have not been quantified, since they are not critical to demonstrating that a weaker gear magnet reduces the spacing between the external and internal magnet arrays. Indeed, the optimal gear separation distance between the two magnet arrays is reduced from 10 to 6 mm without a loss of RMS voltage (Figure 8), thereby substantially reducing the VEH volume.

### 3.2. Optimized VEH

The VEH with a linear MG has been optimized for the number of external weakened magnets and the distance between the two magnet arrays. The fabricated VEH with MG (Figure 9) has the design parameters listed in Table 1.

Mimicking human motion, the VEH with a linear MG is mounted onto a linear actuator, which moves sinusoidally at a certain frequency and acceleration, so that there may be relative velocity between the internal magnets (suspended with ferrofluid bearings inside a chamber) and the coil arrays (mounted outside of the chamber’s bottom surface). At 0.4 g and 3 Hz, the relative velocity of the internal magnets with respect to the VEH chamber is measured from high-speed camera videos. The peak relative velocity from the VEH with a linear MG is almost 1.5 times that from the VEH without an MG (Figure 10) because of the gear effect. The measurements in Figure 10 give Gv around 1.5 at 0.4 g and 3 Hz: the MG raises the peak relative velocity between the internal magnet array and the coil-bearing chamber by about 50%, under the same applied acceleration amplitude or frequency.

The non-zero power generation time per cycle is measured from the open-circuit voltage waveform captured on an oscilloscope. The percentage of the non-zero relative velocity time is greatly increased by the MG, reaching 100% before 3.5 Hz (Figure 11). The improvement in the non-zero relative velocity time over the VEH without an MG is greater at a lower frequency, as the non-zero power generation time is related to the maximum chamber velocity, which is higher at lower frequency for the VEH without MG for a given acceleration.

With 0.4 g acceleration, the VEH with the stronger external magnets (placed at 10 mm above) produces 27.8–66.6 mVrms over 0.9–4.0 Hz with a 127% increase at 4 Hz over a VEH without the MG (Figure 5). With a lower acceleration, 0.3 g, the VEH with the stronger external magnets (placed 10 mm above) produces 26.3–61.6 mVrms over 0.9–4.0 Hz with a 117% increase at 4 Hz over a VEH without the MG (Figure 12). The voltage increase at 0.3 g is not as high as that at 0.4 g, since the VEH with MGs has been optimized for 0.4 g.

## 4. Discussion

With partial demagnetization and a reduced distance between magnet arrays, at 0.4 g acceleration, the VEH with the weaker external magnets (placed at 6 mm above) produces 27.5–62.6 mV_rms_ over 0.9–4.0 Hz with a 114% increase at 4 Hz over a VEH without the MG (Figure 13). The voltage itself and the voltage increase are not as high as those from the VEH with stronger MG, since the gear effect is also reduced, but the volume is reduced by 5%. The figure-of-merit (FOM) is defined as the maximum power delivered to a matched load per the VEH volume per the square of the applied acceleration amplitude, and the FOMs of two different VEHs with MG are presented in Figure 14. The measured open-circuit RMS voltage ($V_{rms}^{2})$ provides the information on the maximum deliverable power to a matched load without requiring another measurement, since a load draws the maximum power of $P_{max}\;=\frac{V_{rms}^{2}}{4R_{L}}$, when the load’s impedance (or resistance $R_{L}$) is equal to the source resistance (or the coil resistance in this case). For the highest measured open-circuit voltage (61.6 mV_rms_) with the 525 Ω coil in the VEH, the corresponding maximum deliverable power is 1.81 µW. This maximum deliverable power is a calculated estimate derived from the open-circuit voltage measurement, not a value obtained from a direct load measurement, so that we may compare the power density of different VEHs. The FOMs of VEHs with different strengths of LMG are similar, with differences of less than 5%.

Compared with other publications on electromagnetic VEHs, including our earlier ones [12,13], this paper is the only one that addresses sub-Hz and sub-g human motion by electromagnetic energy harvesting under 3 cc (Table 2). Our current VEH, with a volume of 2.5 cc, generates μW-level power and is the only one to employ a linear magnetic gear mechanism to increase the harvested power, albeit much more effectively for >3 Hz acceleration.

The measurements in this paper have been obtained with sinusoidal motion on a linear actuator at various frequencies and accelerations in order to present quantitative improvement with the MG, since the MG’s benefit varies with frequency and amplitude (Figure 5, Figure 6, Figure 7, Figure 8, Figure 9, Figure 10, Figure 11, Figure 12 and Figure 13). Thus, though the presented results are on the specific sinusoidal frequencies and accelerations (in terms of the measured RMS voltages, relative velocities, and improvements due to the linear magnetic gear), the impact of the results on real, irregular, multi-directional, multi-frequency human motion can be predicted, as the VEH is (1) a non-resonant vibrational energy harvester that can generate power from vibration/acceleration of multiple frequencies [12] and (2) small enough to be made into an array of three with each one taking care of the orthogonal directions (i.e., x, y, and z directions).

## 5. Conclusions

This paper presents a non-resonant electromagnetic VEH integrated with a linear magnet array to enhance the relative velocity between the internal magnet array and the coil array, thereby improving power generation from sub-g acceleration. The linear magnetic gear is optimized as a function of the number of external magnets, the distance between the external and internal magnets, and the magnetic field strength of the external magnets, for a non-resonant VEH for sub-g acceleration, associated with human daily motions. The MG’s RMS voltage improvement increases markedly above 3 Hz, the critical frequency, where the dead-zone fraction is already small, in the movable ranges of the devices presented in this paper. A longer movable range would lower the critical frequency and extend the marked improvement to below 3 Hz, at the cost of device volume.

## Figures and Tables

**Figure 1:**
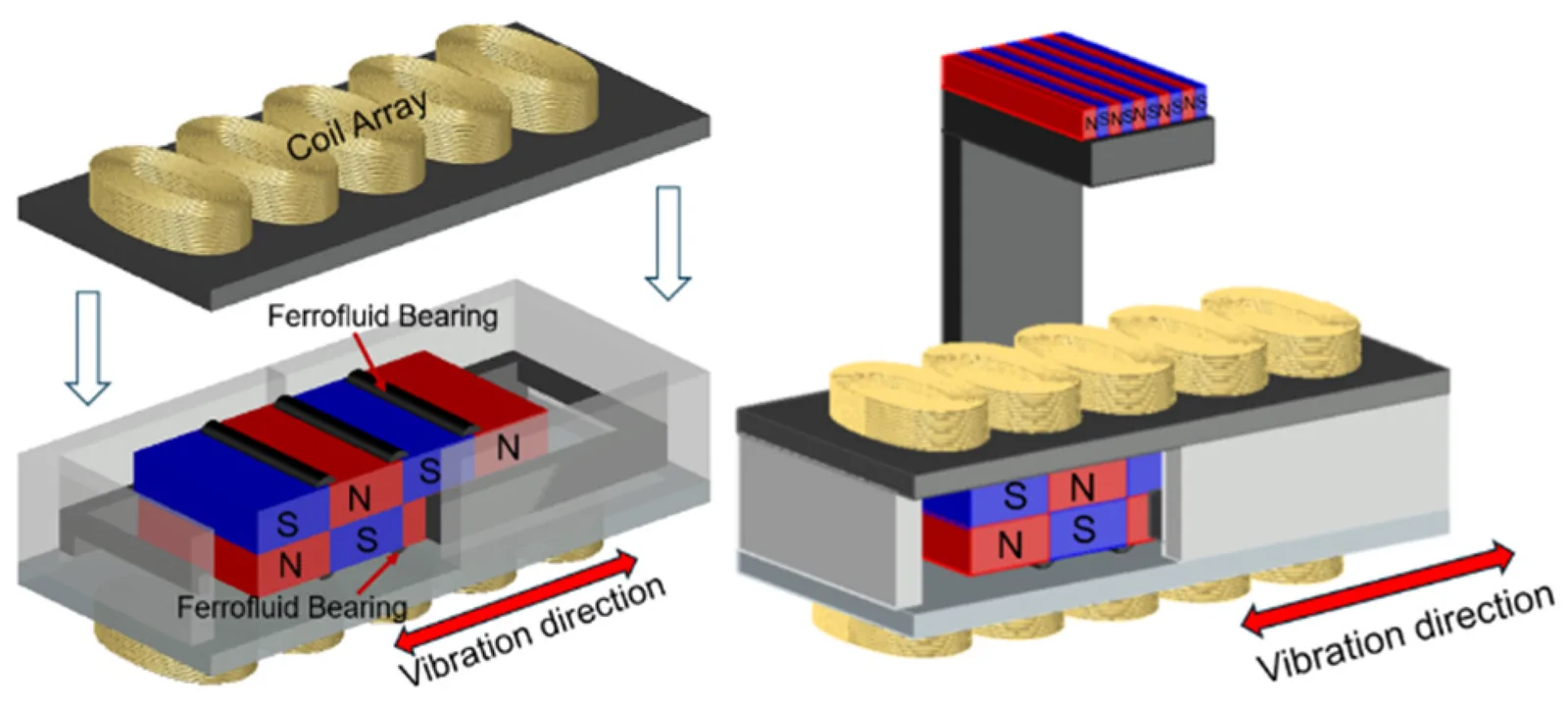
Schematics of the vibration energy harvesters (VEHs) with the magnetic array suspended by ferrofluid bearings at the top and bottom of the array, without (**left**) and with (**right**) linear magnetic gear (MG).

**Figure 2:**
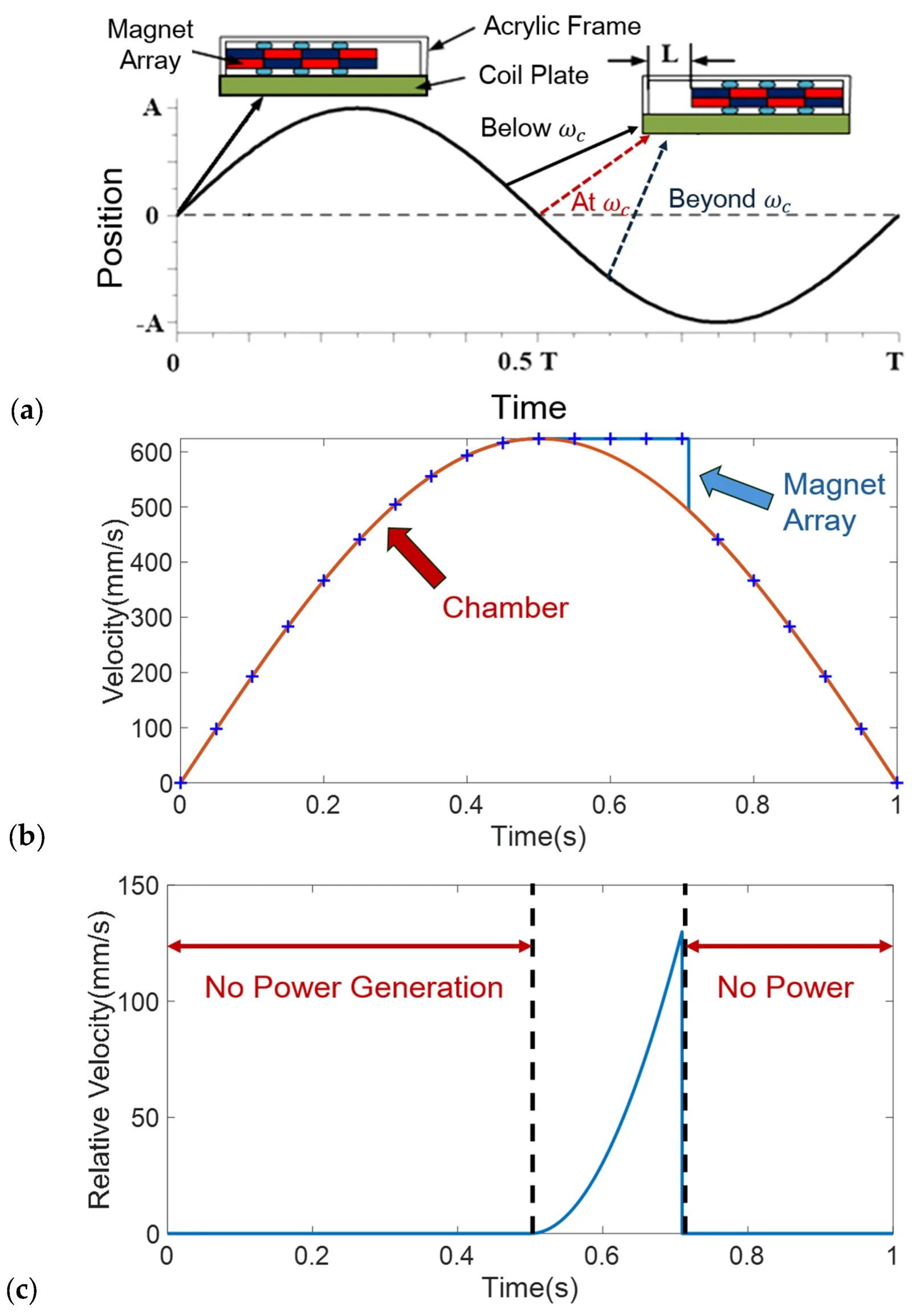
Movement of the VEH without magnetic gears vs. time in response to an applied 0.5 Hz, 0.2 g sinusoidal acceleration; positions and velocities are computed from a kinematic frictionless model; (**a**) positions of the VEH chamber and internal magnet array, (**b**) velocities of the chamber and internal magnet array in a half cycle, and (**c**) relative velocity between the chamber and internal magnet array showing non-zero relative velocity (needed for power generation) for only 21% of the time.

**Figure 3:**
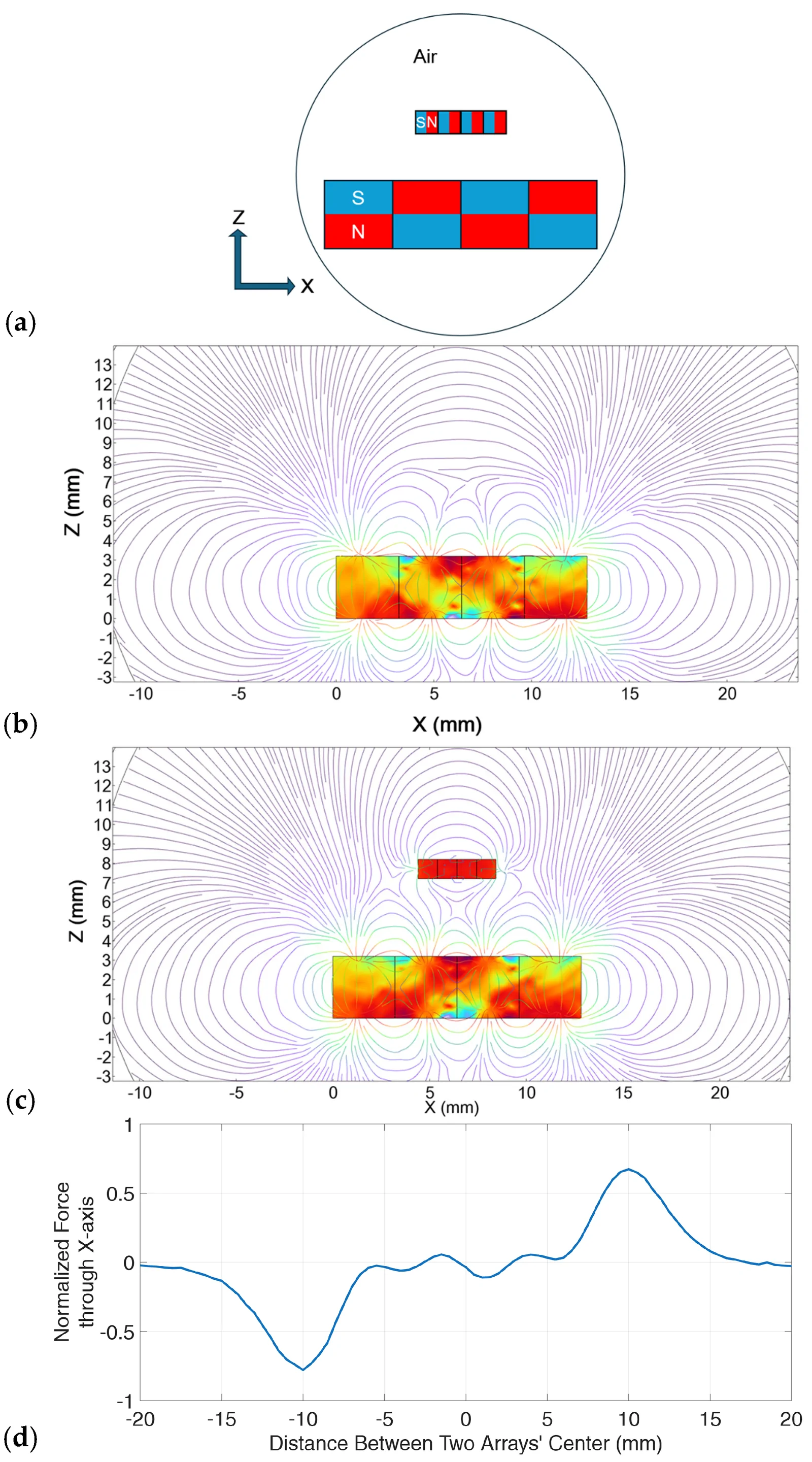
COMSOL simulation of the two arrays of magnets of different sizes (**a**), showing the magnetic field’s distribution around the bigger-size magnets without (**b**) and with (**c**) smaller-size magnets. The *x*-axis force from the smaller magnets helps the larger magnets gain greater velocity (**d**).

**Figure 4:**
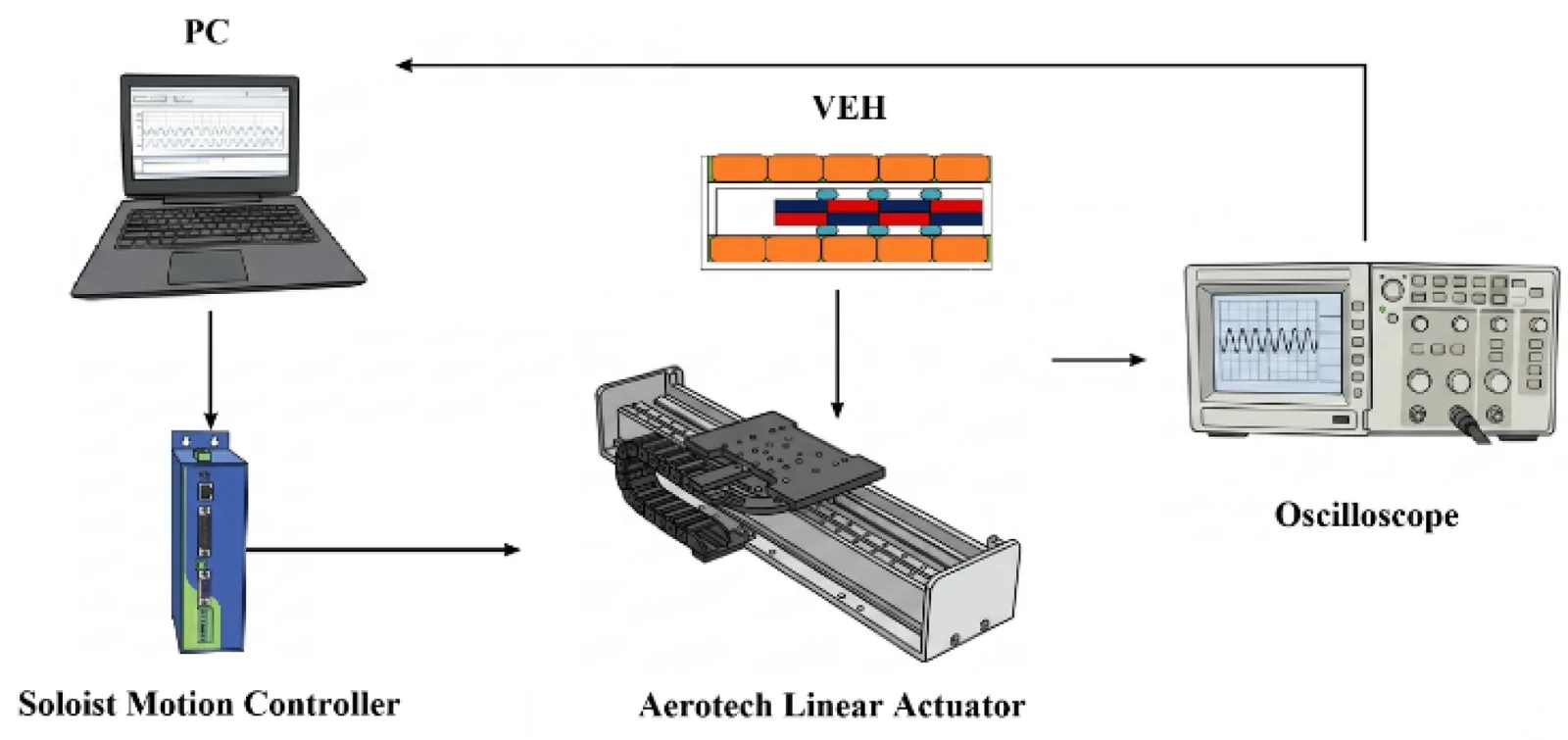
Experimental setup for VEH characterization.

**Figure 5:**
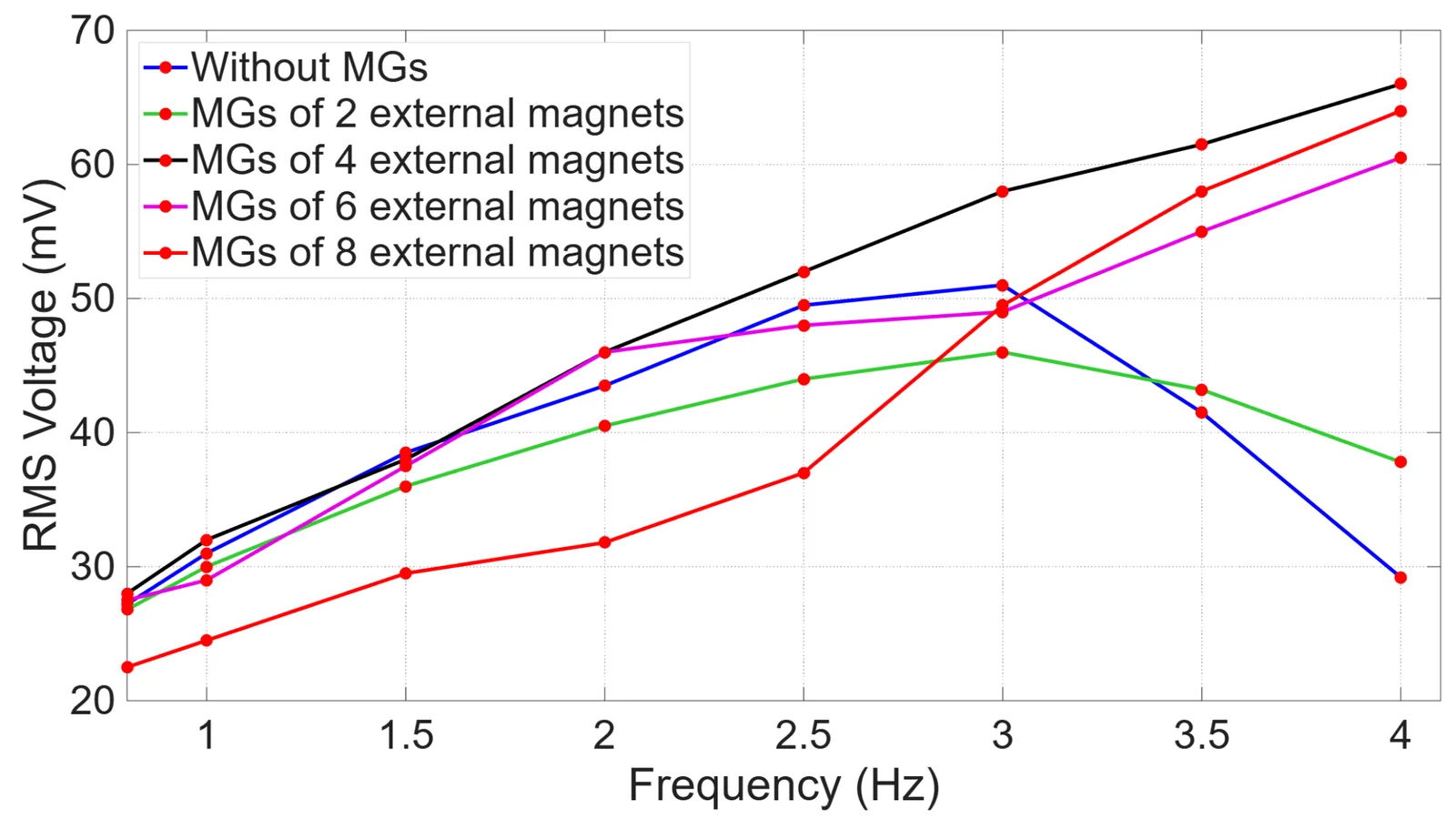
Measured open-circuit RMS voltage vs. vibration frequency without and with the magnetic gears of 2–8 magnets for a 0.4 g acceleration.

**Figure 6:**
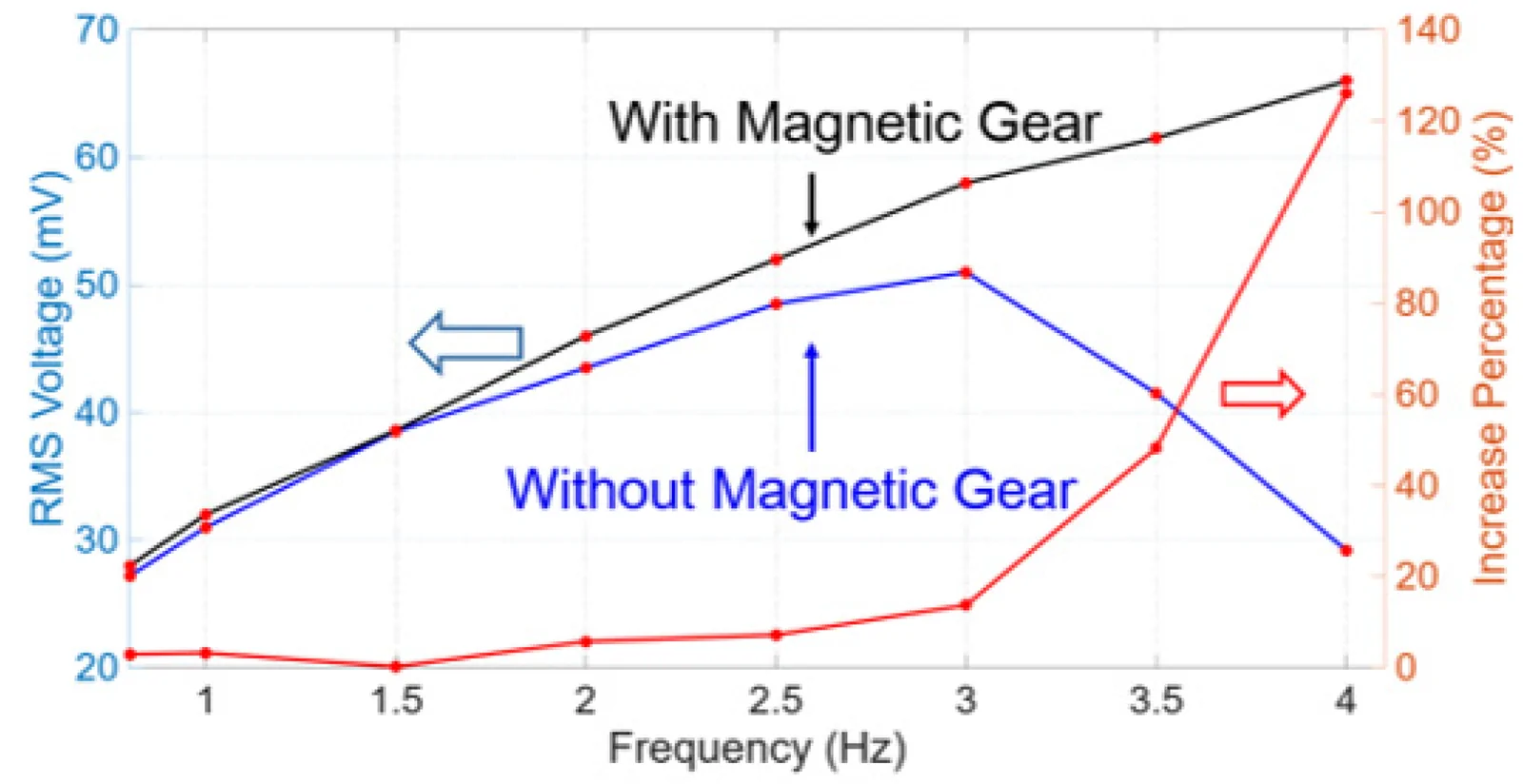
Measured RMS voltage vs. vibration frequency with and without the magnetic gear at 0.4 g acceleration, showing a 127% RMS voltage increase at 4 Hz.

**Figure 7:**
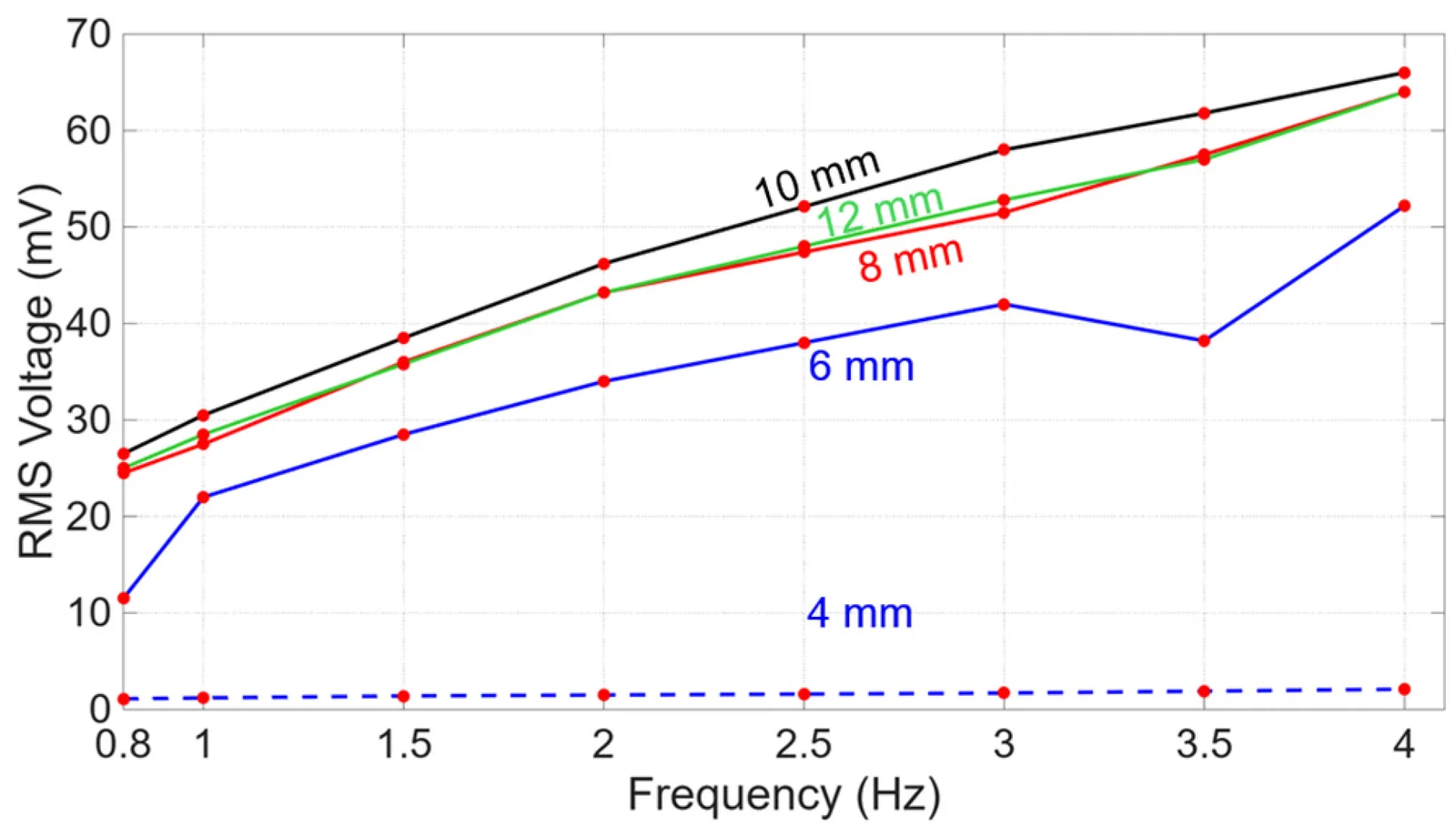
Measured open-circuit RMS voltages vs. vibration frequency as a function of the distance between the internal magnet array and external magnet array with stronger external magnets.

**Figure 8:**
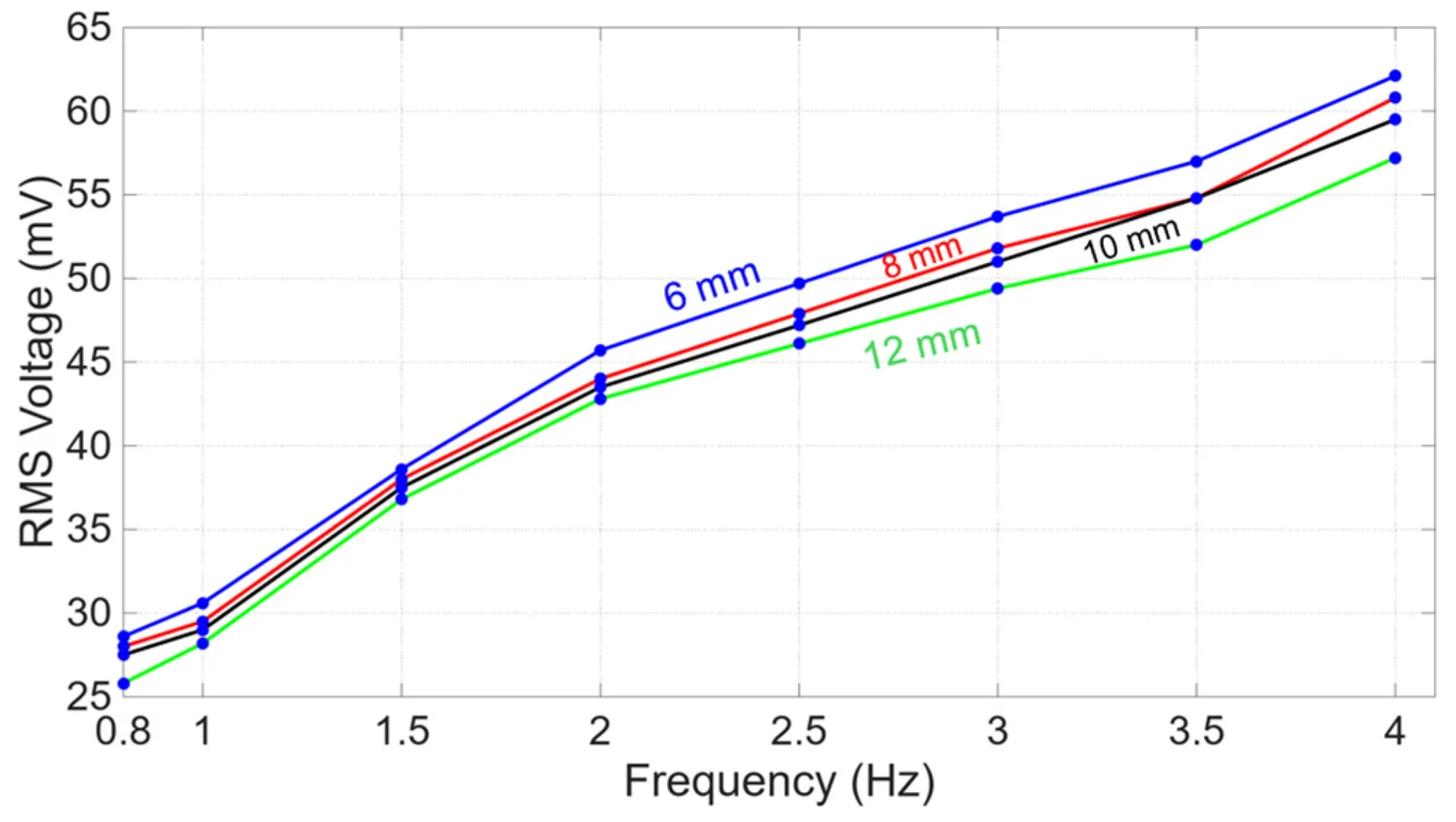
Measured open-circuit RMS voltages vs. vibration frequency as a function of the distance between the internal magnet array and external magnet array with weaker external magnets.

**Figure 9:**
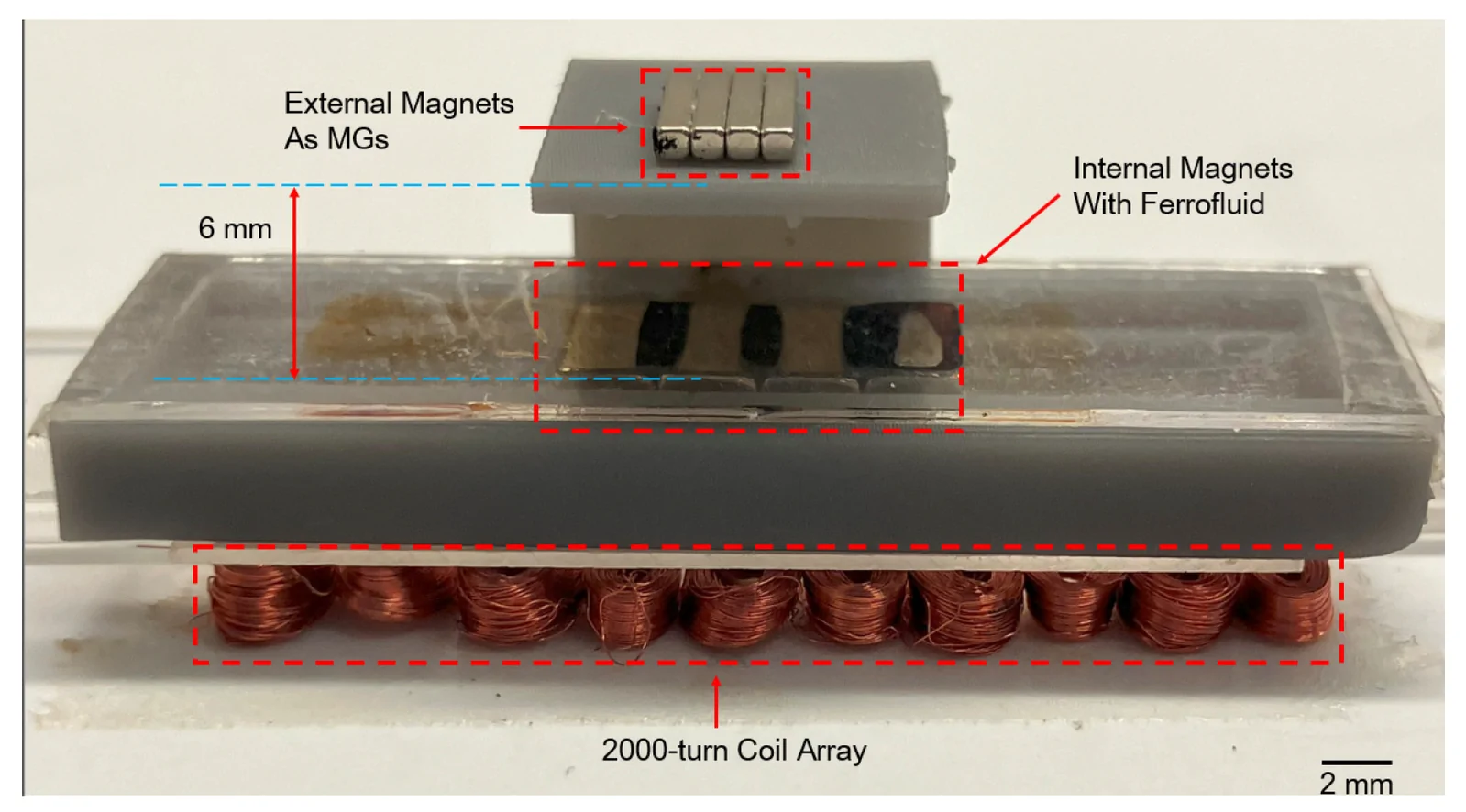
Photograph of the VEH with the magnetic gear configuration listed in Table 1.

**Figure 10:**
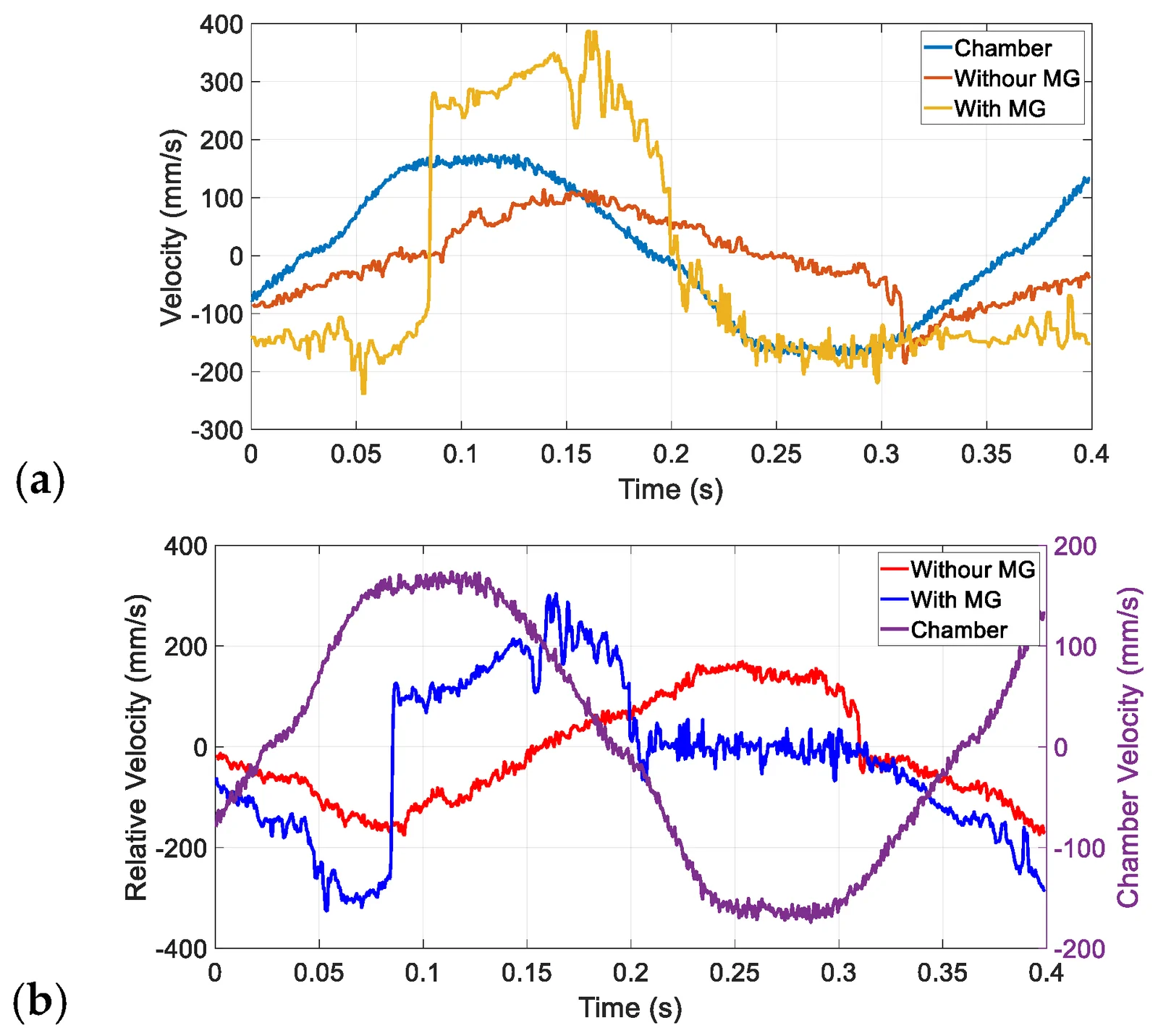
(**a**) Measured velocities vs. time at 0.4 g and 3 Hz. (**b**) Measured relative velocities vs. time at 0.4 g and 3 Hz, showing the velocity increase due to the gear effect with 4 external magnets as a magnetic gear.

**Figure 11:**
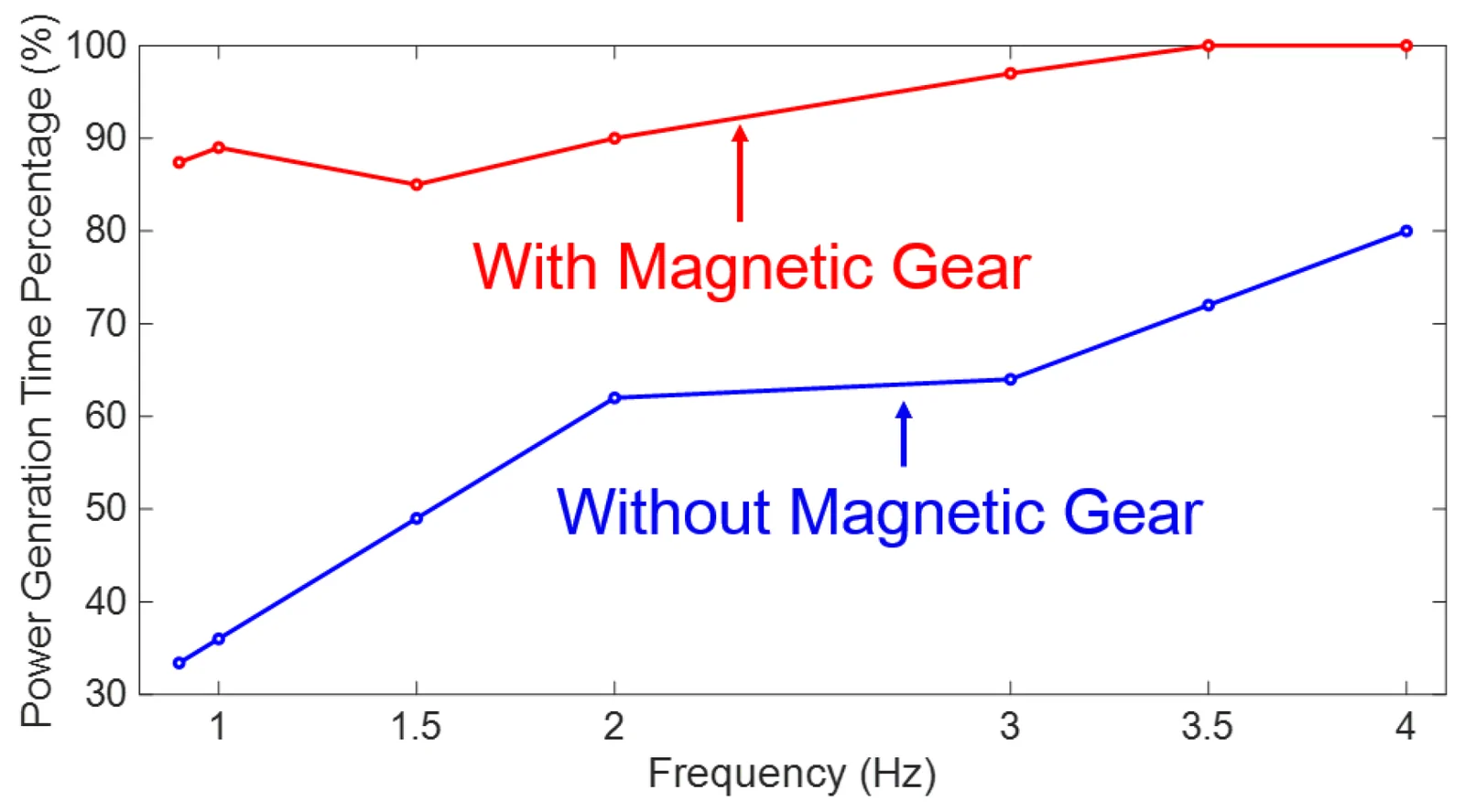
Measured percentage of non-zero relative velocity time vs. vibration frequency with and without the magnetic gear (with four external magnets) at 0.4 g acceleration, showing the gear effect enhancing the duration of non-zero relative movement (between the internal magnet array and the chamber that houses the coil array) that is needed for power generation.

**Figure 12:**
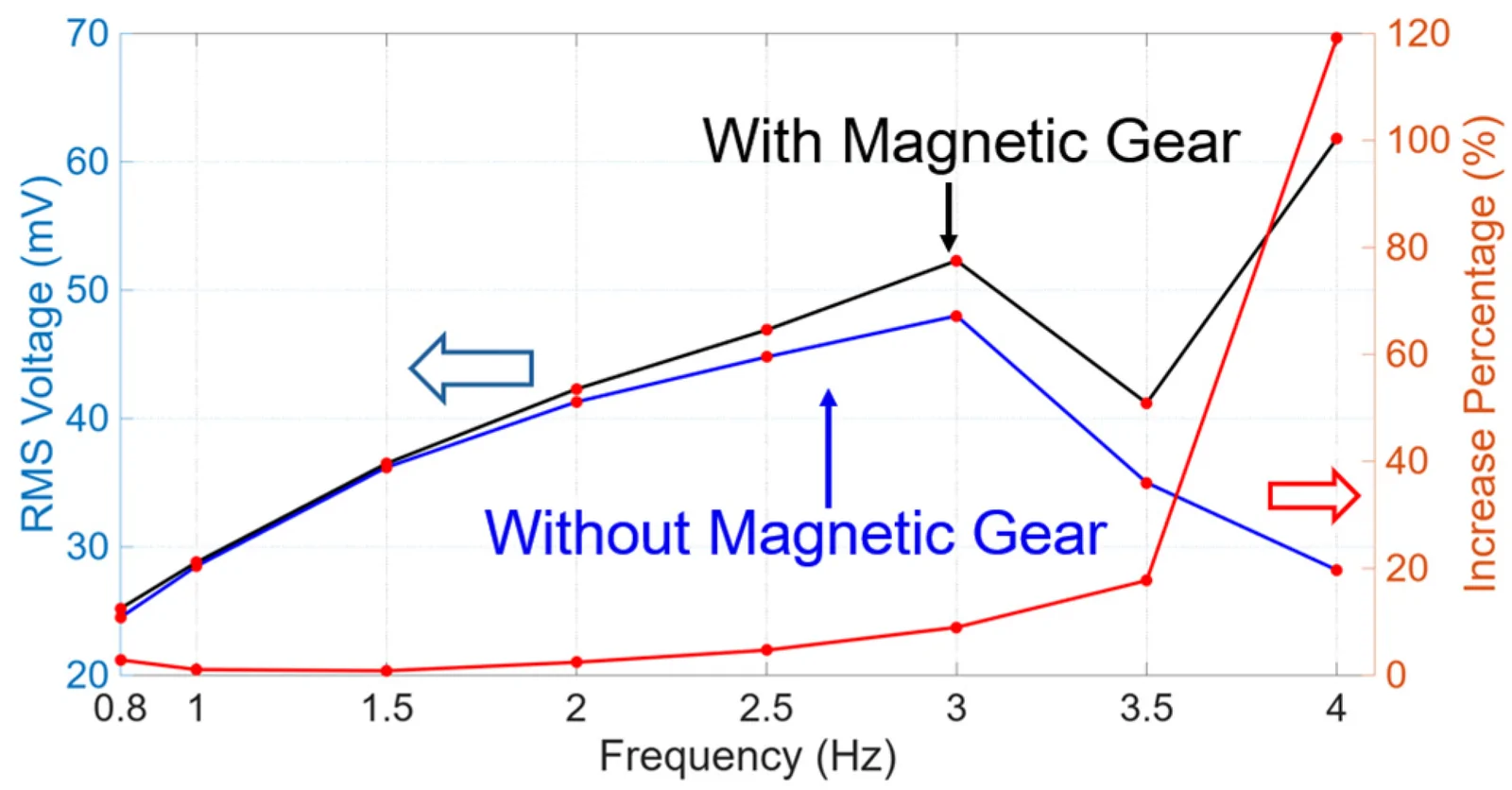
Measured RMS voltage vs. vibration frequency with and without the magnetic gear with stronger magnets at 0.3 g acceleration, showing a 117% voltage increase at 4 Hz.

**Figure 13:**
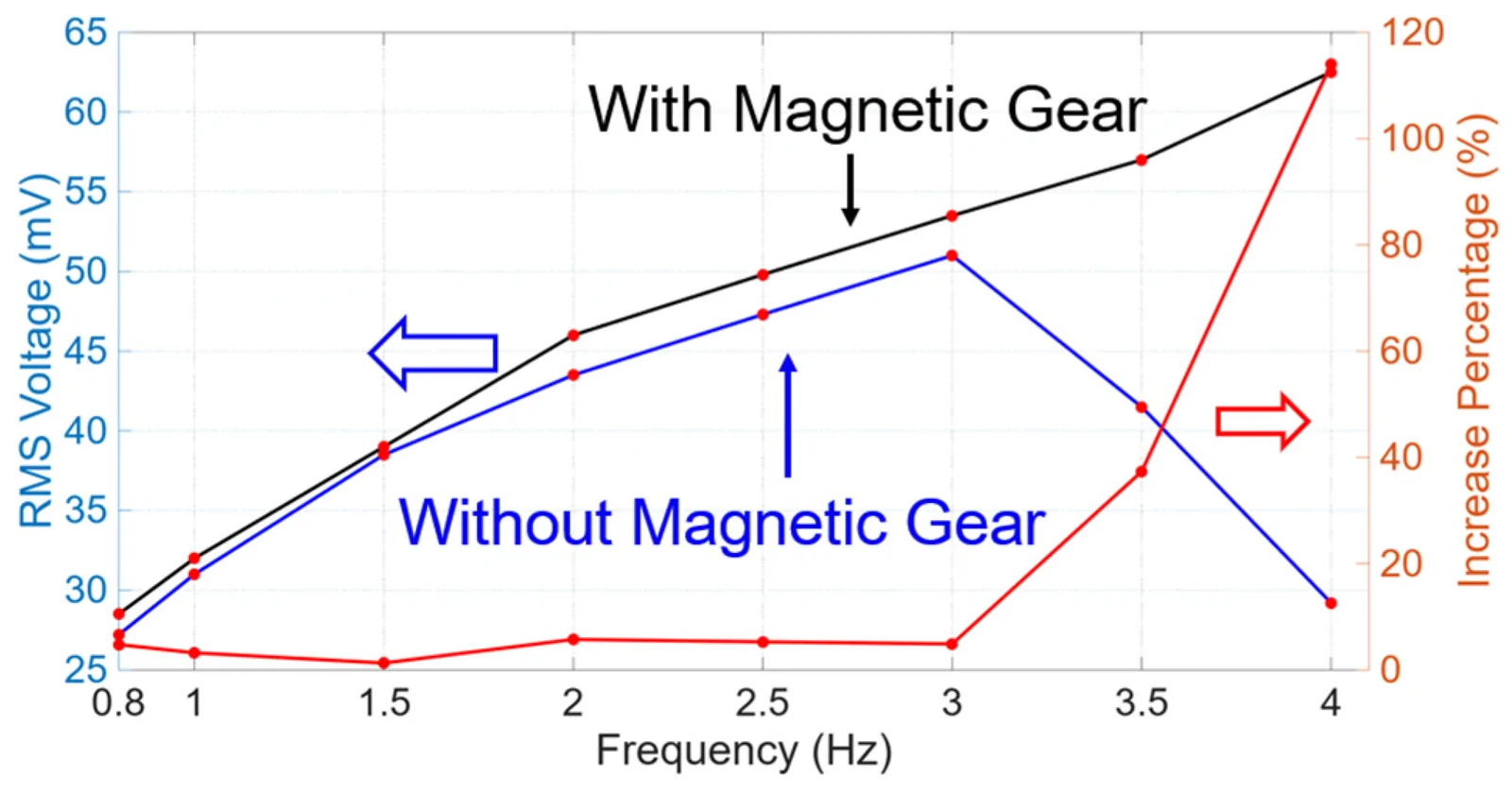
Measured RMS voltage vs. vibration frequency with and without the magnetic gear with weaker magnets at 0.4 g acceleration, showing a 114% voltage increase at 4 Hz.

**Figure 14:**
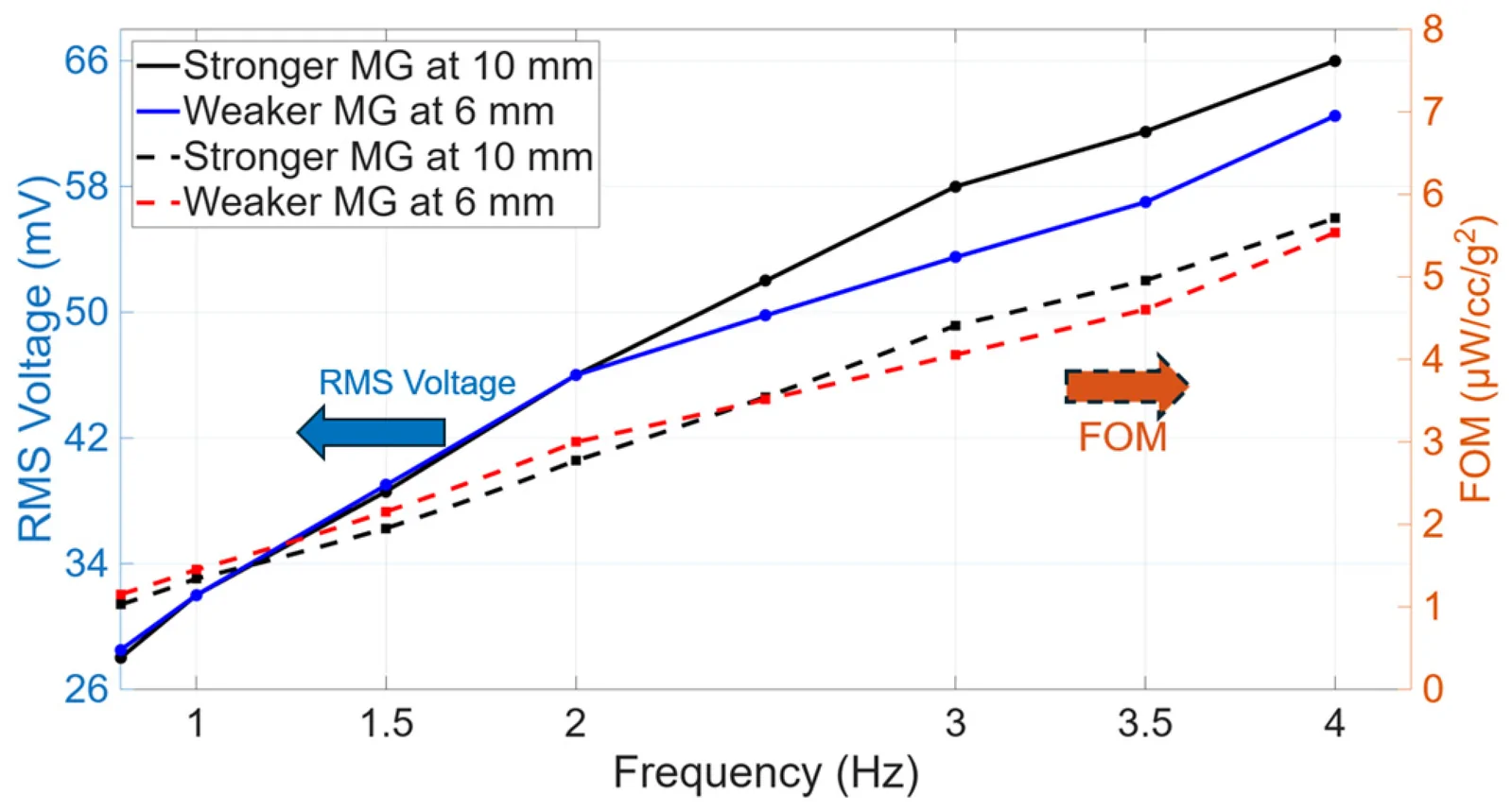
Measured FOM vs. vibration frequency with MGs having different strengths under 0.4 g acceleration, showing similar FOM.

**Table 1:** Optimized non-resonant VEH with a linear magnetic gear composed of external weakened magnets.

Total Volume and Weight	2.5 cc and 5.5 g
Internal Magnet Size and Number	3.2 × 6.4 × 3.2 mm^3^ and 4
External Magnet Size and Number for MG	1.0 × 6.4 × 1.0 mm^3^ and 4
Movable Range of Internal Magnet Array	25.0 mm
Chamber Size (mm^3^)	37.8 × 6.8 × 3.8
Coil Array Size (mm^3^)	7.0 × 3.4 × 2.2
Total Coil Turns	2000
Total Coil Resistance	525 Ω

**Table 2:** Comparison of other works of electromagnetic VEH on human wearables.

Reference	Volume (cc)	Frequency (Hz)	Acceleration (g)	Power (mW)
[12]	1.1	2	2	0.012
[13]	1.4	1	0.2	0.0009
[17]	6.5	5.2	2.1	2.15
[18]	34.8	41.2	1	3.71
[19]	5.9	63.7	1	1.18
[20]	32.8	11	0.5	1.1
[21]	12	9	0.8	1.15
[10]	119	4	6.4	87.1
This work	2.5	0.8	0.4	0.0006

## Data Availability

Data are contained within the article.
